# Computational systems biology for omics data analysis

**DOI:** 10.1093/jmcb/mjz095

**Published:** 2019-10-09

**Authors:** Luonan Chen

**Affiliations:** Key Laboratory of Systems Biology, Center for Excellence in Molecular Cell Science, Shanghai Institute of Biochemistry and Cell Biology, Chinese Academy of Sciences, Shanghai, China

Recent trend on biological data at a molecular level is omics data analysis for both bulk and single cells, including genomics, proteomics, metabolomics, and epigenetics data (Wang and Zhang, 2017; Zhang et al., 2017; Zhao and Li, 2017; Cheng and Leung, 2018). Rapid accumulation of such high-dimensional biological data is driving the system-level study from describing complex phenomena to understanding molecular mechanisms (Park et al., 2018; Sun et al., 2018) and from analyzing individual components to understanding their networks and systems (Chen et al., 2009; Chen, 2017). Omics data analysis from the perspective of computational systems biology is increasingly attracting the attention from computational biology community, which aims to provide essential tools for gaining new insights into biological processes or systems (Zhang et al., 2015; Sa et al., 2016; Li et al., 2017; Liu et al., 2019a, b). In this issue, we collect six research articles and one Perspective, which are all related to such high-dimensional omics data analysis, ranged from new concepts of biomarkers (network biomarker for disease diagnosis and dynamic network biomarker (DNB) for disease prediction) to single-cell sequencing analyses, to neuron science and disease analyses. These papers were mainly from the contributors to The 12th International Conference on Computational Systems Biology (ISB 2018).

The cerebellum is critical for controlling motor and nonmotor functions via cerebellar circuit that is composed of defined cell types, which account for more than half of neurons in mammals. Luo’s laboratory analyzed transcriptome profiles of 21119 single cells of the postnatal mouse cerebellum and identified eight main cell clusters. Functional annotation of differentially expressed genes revealed trajectory hierarchies of granule cells at various states and implied roles of mitochondrion and ATPases in the maturation of Purkinje cells, the sole output cells of the cerebellar cortex. This study presents a systematic landscape of cerebellar gene expression in defined cell types and a general gene expression framework for cerebellar development and dysfunction. Furthermore, Dr Ying Shen highlighted that this work accelerates the understanding of molecular and cellular mechanisms of cerebellar circuitry and diseases, and thus may help to design more specific interventions in the future.

Based on nonlinear dynamics theory, the DNB method (Liu et al., 2019a, b) is designed to quantitatively identify the tipping point of a drastic system transition and can theoretically identify DNB genes that play key roles in acquiring drug resistance. Liu et al. (2019a) analyzed time-course mRNA sequence data generated from the tamoxifen-treated estrogen receptor-positive MCF-7 cell line and identified the tipping point of endocrine resistance with its leading molecules. The results show that there is interplay between gene mutations and DNB genes, in which the accumulated mutations eventually affect the DNB genes that subsequently cause the change of transcriptional landscape, enabling full-blown drug resistance. On the other hand, developing effective and non-invasive biomarkers of hepatocellular carcinoma (HCC) for individual patients remains an urgent task for early diagnosis and convenient monitoring. By analyzing the transcriptomic profiles of peripheral blood mononuclear cells from both healthy donors and patients with chronic hepatitis B virus (HBV) infection in different states, Hu, Chen, and Su’s laboratories collaborated to identify 19 candidate genes according to network biomarkers (Zhang et al., 2015; Liu et al., 2016) and DNB (Liu et al., 2019a, b). The study not only provides a novel network biomarker or edge-based biomarker for non-invasive and effective diagnosis of HBV-associated HCC to each individual patient but also introduces a new way to integrate the interaction terms of individual molecules for clinical diagnosis and prognosis from the network and dynamics perspectives.

Analysis linking directly genomics, neuroimaging phenotypes, and clinical measurements is of great importance for understanding psychiatric disorders. Ma and colleagues described a multiscale analysis method using genome-wide single-nucleotide polymorphisms, gene expression, gray matter volume, and the positive and negative syndrome scale scores to explore the etiology of schizophrenia. The results show how genetic variants may affect brain structures that lead to distinct disease phenotypes. Also, the method of multiscale analysis described in this research may help to advance the understanding of the etiology of schizophrenia. On the other hand, the set of genes or proteins required for mitotic division remains poorly characterized. Freeman’s group systematically conducted an extensive series of correlation analyses of human and mouse transcriptomics data to identify > 700 cell cycle-associated genes strongly and reproducibly associated with cells undergoing S/G2-M phases of the cell cycle. This study presents the first comprehensive list of human cell cycle proteins, identifying many new candidate proteins.

The specific mechanism underlying synaptic plasticity remains unclear. Wen’s group observed abnormal neural and dendritic morphology in the hippocampus following knockout of *Atp11b* both *in vitro* and *in vivo*. The experimental results also indicated that ATP11B regulated synaptic plasticity in hippocampal neurons through the MAPK14 signaling pathway. This work sheds light on the possible mechanisms underlying the regulation of synaptic plasticity and laid the foundation for the exploration of proteins involved in signal transduction during this process.

At last, Veitia comprehensively reviewed the topics related to selective Darwinian advantage trends as a Perspective, which showed that the replacement of a cell type by another bearing a selective advantage can be conveniently modeled by a logistic function according to different formalisms and assumptions. Furthermore, given the continuous nature of the population replacement process, Veitia showed that the replacement curve should be sigmoidal whether or not the underlying function is in the form of the classical logistic equation.
